# Comparison Between Systemic Lupus Erythematosus Patients with and Without Family History of Systemic Lupus Erythematosus—A Single-Center Retrospective Study

**DOI:** 10.3390/jcm15197481

**Published:** 2026-09-25

**Authors:** Joanna Kosałka-Węgiel

**Affiliations:** 1 Department of Rheumatology and Immunology, Jagiellonian University Medical College, Jakubowskiego 2, 30-688 Kraków, Poland; joanna.kosalka@uj.edu.pl; Tel.: +48-12-400-31-10; 2 Department of Rheumatology, Immunology and Internal Medicine, University Hospital in Kraków, Jakubowskiego 2, 30-688 Kraków, Poland

**Keywords:** clinical phenotype, family history, first-degree relatives, systemic lupus erythematosus

## Abstract

**Background**: A positive family history of systemic lupus erythematosus (SLE) reflects the substantial genetic contribution to disease susceptibility; however, it remains unclear whether patients with a family history of SLE represent a distinct clinical phenotype. **Methods**: A retrospective single-center study was conducted in adult patients fulfilling the 2019 EULAR/ACR classification criteria for SLE who were treated at the University Hospital in Kraków, Poland, between 2012 and 2022. Demographic, clinical, laboratory, comorbidity, and treatment data, as well as mortality data, were extracted from medical records. Patients with and without a documented/reported family history of SLE were compared, and those with positive family history of SLE were further stratified according to the degree of kinship of the affected relative. Patients with SLE for whom data on a family history of SLE were unavailable were not included in the analysis. Benjamini–Hochberg false discovery rate correction was applied across the primary comparisons, while the kinship analysis was corrected separately. **Results**: Among 723 patients with SLE, 47 (6.5%) reported a family history of SLE. Patients with a family history of SLE had a similar overall demographic and clinical profile to those without a family history. Arthralgia was nominally more frequent in patients with a family history of SLE (97.8% vs. 88.5%, *p* = 0.049), but this association did not remain significant after correction for multiple comparisons (*q* = 1.000). Similarly, myocardial infarction was more frequent in patients with a family history of SLE (7/47 [14.9%] vs. 37/676 [5.5%], *p* = 0.019), but the association was not significant after FDR correction (*q* = 0.912). No significant differences were observed in the serological profile, antiphospholipid antibody prevalence, treatment exposure, or mortality after correction for multiple comparisons. In the kinship analysis, several nominal differences in clinical manifestations were observed between patients with affected first-degree and more distant relatives, including arthritis, malar rash, oral/nasal ulcers, and thrombocytopenia; however, none remained statistically significant after correction for multiple comparisons. **Conclusions**: A positive family history of SLE was reported by a small proportion of patients in this European cohort and was not associated with a distinct overall clinical, serological, or treatment profile after correction for multiple comparisons. Nominal associations with selected clinical manifestations and myocardial infarction, as well as differences observed according to the degree of kinship, should be interpreted cautiously given the small familial subgroup and multiple comparisons. These findings are hypothesis-generating and require confirmation in larger prospective multicenter cohorts.

## 1. Introduction

Systemic lupus erythematosus (SLE) is a chronic autoimmune disease characterized by the production of pathogenic autoantibodies, immune complex deposition, inflammation, and progressive organ damage resulting from the loss of immune tolerance [1]. Its pathogenesis involves a complex interplay of genetic susceptibility, environmental triggers, and immune dysregulation, including defective clearance of apoptotic cells and immune complexes together with aberrant activation of the type I interferon pathway [2].

Among these, genetic predisposition plays a central role in disease susceptibility [3,4,5]. SLE is a highly polygenic disorder, with genome-wide association studies (GWAS) identifying more than 120 susceptibility loci, most of which are located in non-coding regulatory regions and individually exert only modest effects on disease risk [3,4,6,7]. Rather than resulting from a single causative mutation, SLE develops through the cumulative effects of numerous common genetic variants that collectively disrupt immune regulation and predispose individuals to autoimmunity [3,6,7]. Among these susceptibility loci, variants within the major histocompatibility complex (MHC) on chromosome 6 represent the strongest genetic risk factors, while inherited deficiencies in complement components further increase susceptibility to the disease [8,9,10]. Notably, many of these genetic risk loci have been consistently replicated across diverse ethnic populations, although population-specific variants may contribute to differences in disease susceptibility and the clinical heterogeneity characteristic of SLE [5,11]. These findings highlight the strong hereditary component and polygenic nature of the disease [5,11].

A positive family history of SLE refers to the presence of SLE in one or more relatives of an affected individual [12]. The reported prevalence of a positive family history of SLE varies widely, reaching up to 50%, depending on clinical manifestations, ethnicity, and age at disease onset [13,14,15]. Such high estimates may partly reflect relatively small study populations and differences in the characteristics of the included cohorts. Moreover, some of the earlier studies were conducted before the introduction of the currently used SLE classification criteria, which may have contributed to differences in patient selection and reported prevalence [15]. However, the available evidence remains inconsistent, and relatively few studies have comprehensively characterized the clinical features of patients with a family history of SLE [13,14,15]. Moreover, it remains unclear whether a positive family history of SLE is associated with a distinct clinical phenotype or differences in the recorded clinical manifestations of the disease. In addition, most published data are derived from Asian and North American populations, whereas evidence from European cohorts remains limited [13,15,16].

A better understanding of the clinical characteristics of patients with a family history of SLE may provide insight into the relationship between genetic susceptibility and disease expression, facilitate earlier recognition of the disease among at-risk relatives, and support more personalized patient management.

Therefore, this study aimed to evaluate the clinical characteristics, serological profiles, and treatment patterns of patients with a family history of SLE and to determine whether disease presentation and recorded clinical manifestations differ between patients with affected first-degree relatives and those with only more distant affected relatives.

## 2. Materials and Methods

### 2.1. Study Population

This retrospective single-center study included patients diagnosed with SLE and treated at the University Hospital in Kraków, Poland, between 2012 and 2022. Eligible patients fulfilled the 2019 European Alliance of Associations for Rheumatology (EULAR)/American College of Rheumatology (ACR) classification criteria for SLE [17]. The 2019 EULAR/ACR classification criteria were applied retrospectively to all patients to ensure a consistent and homogeneous SLE study population and to allow classification according to the currently recommended international criteria.

Demographic, clinical, and laboratory data were extracted from medical records. Collected variables included sex, age, age at symptom onset, age at diagnosis, disease duration, comorbidities, treatment received, and, where applicable, mortality. Clinical manifestations of SLE were recorded as ever/never variables, indicating whether a given manifestation had been documented at any time during the available follow-up after SLE diagnosis, rather than only at the time of diagnosis. Clinical manifestations evaluated comprised mucocutaneous involvement, arthritis, serositis, hematologic abnormalities, renal, hepatic, neurological, and pulmonary involvement, Raynaud’s phenomenon, and lymphadenopathy [18]. Immunosuppressive treatments were also recorded as ever/never variables, based on whether a patient had received a given treatment at any time during the available follow-up. Comorbidities were recorded if documented at any time in the available medical record, regardless of whether they occurred before or after the diagnosis of SLE.

The primary variable of interest was a documented or reported family history of SLE. Information on family history was obtained from patient self-report and/or available medical records. Patients with a documented/reported positive family history of SLE were classified as having a family history of SLE, whereas patients with a documented/reported negative family history were classified as having no family history of SLE. Patients for whom information on family history of SLE was unavailable were excluded from the analysis. Among patients with a documented/reported family history of SLE, affected relatives were categorized according to the degree of kinship as first-degree or second-degree (or more distant) relatives. In patients reporting more than one affected relative, classification was based on the closest degree of kinship; thus, patients with both first-degree and more distant affected relatives were classified according to their first-degree relative.

### 2.2. Laboratory Analysis

Routine laboratory investigations included complete blood count, urinalysis with sediment examination, and 24-h urinary protein excretion. Antinuclear antibodies (ANA) were detected by indirect immunofluorescence (IIF) on HEp-2 cells, followed by enzyme-linked immunosorbent assay (ELISA) or line immunoblot assays for the identification of specific autoantibodies. Anti-double-stranded DNA (anti-dsDNA) antibodies were determined using the *Crithidia luciliae* immunofluorescence assay. Hypercoagulability testing included lupus anticoagulant as well as immunoglobulin (Ig)G and IgM anti-cardiolipin and anti-β2-glycoprotein I antibodies.

### 2.3. Statistical Analysis

Analyses were conducted in R 4.5.3 (R Core Team, 2026). The analysis included patients with available information on family history of SLE. Categorical and continuous variables were summarised as frequencies (*n*/N, %) and medians (Q1–Q3), respectively. Patients with and without a family history of SLE were compared using Fisher’s exact test and the Wilcoxon rank-sum test, respectively. For binary variables, we report the prevalence odds ratio (OR) with 95% confidence interval (CI) and the difference in prevalence (percentage points) with 95% CI; odds ratios are not estimable when one group contained no events. To account for false discovery, the Benjamini–Hochberg (BH) procedure was applied across all 97 primary covariate comparisons, rather than separately within individual clinical domains. Both crude *p*-values and BH-adjusted *q* values are reported. The additional analysis comparing patients with affected first-degree relatives with those with more distant affected relatives was treated as a separate family of 84 comparisons, and the BH procedure was applied separately to these comparisons. Because of the small number of events, no multivariable adjustment was performed and all estimates, including that for MI, are unadjusted. Tests were two-sided; *q* < 0.05 was considered statistically significant, and a *p*-value < 0.05 that did not survive correction is referred to as nominally significant.

### 2.4. Ethics

The study protocol was approved by the Bioethics Committee of the Jagiellonian University Medical College (approval No. 118.6120.21.2023, 15 June 2023). The study was conducted in accordance with the principles of the Declaration of Helsinki.

## 3. Results

### 3.1. Demographic Features

Table 1 summarizes the baseline characteristics of patients with SLE according to the presence or absence of a family history of SLE. Among the 723 patients included in the study, 47 (6.50%) reported a family history of SLE. The proportion of female patients was comparable between patients with and without a family history of SLE (85.1% vs. 88.0%, *p* = 0.495). Likewise, no significant differences were observed in current age, age at SLE onset, age at first SLE symptoms, disease duration, or mortality between the two groups (all *p* > 0.05).

### 3.2. Clinical Manifestations in Systemic Lupus Erythematosus Patients with Family History of Systemic Lupus Erythematosus

Table 2 summarizes the cumulative frequencies of clinical manifestations in patients with SLE according to the presence or absence of a family history of SLE. Among patients with a family history of SLE, joint manifestations were observed in 97.9% of patients, while hematological and constitutional manifestations were each present in 87.2% of cases. In contrast, among patients without a family history of SLE, hematological (92.9%), joint (88.2%), and mucocutaneous (81.2%) manifestations were the most frequently observed clinical features.

Comparison of the two groups revealed no significant differences in the frequencies of most clinical manifestations (all *p* > 0.05). Although arthralgia was more frequent among patients with a family history of SLE (97.8% vs. 88.5%, *p* = 0.049), this association did not remain statistically significant after correction for multiple comparisons (*q* = 1.000). Similarly, the frequency of overall joint manifestations was higher in patients with a family history of SLE (97.9% vs. 88.2%, *p* = 0.051; *q* = 1.000). No other clinically relevant differences were observed between the groups.

### 3.3. A Frequency of Medical Comorbidities in Systemic Lupus Erythematosus Patients with Positive Family History of Systemic Lupus Erythematosus

The prevalence of comorbid conditions in patients with SLE according to family history of SLE is summarized in Table 3. Overall, the majority of the analyzed comorbidities were similarly distributed between patients with and without a family history of SLE. Specifically, endocrine disorders (hypothyroidism and hyperthyroidism), end-stage kidney disease (ESKD), cardiometabolic conditions including arterial hypertension, diabetes mellitus, and hypercholesterolaemia, as well as cardiovascular diseases such as heart failure, atrial fibrillation, and peripheral arterial disease, occurred with comparable frequency in both groups (all *p* > 0.05).

Myocardial infarction was more frequent among patients with a family history of SLE than among those without a family history (14.9% vs. 5.5%; OR = 3.02, 95% CI: 1.07–7.44; *p* = 0.019). However, this association did not remain statistically significant after correction for multiple comparisons (*q* = 0.912). The prevalence of malignancy and monoclonal gammopathy of undetermined significance (MGUS) was similar between the groups. Likewise, the frequency of arterial and venous thrombotic events, including stroke, transient ischaemic attack, deep vein thrombosis, and pulmonary embolism, did not differ significantly according to family history of SLE.

### 3.4. Antibody Profiles in Systemic Lupus Erythematosus Patients with Family History of Systemic Lupus Erythematosus

A summary of the autoantibody profile of the analyzed patients is presented in Table 4. The median recorded ANA titre did not differ significantly between patients with and without a family history of SLE (10,240 [2560–20,480] in both groups; *p* = 0.962, *q* = 1.000).

Among patients with a family history of SLE, anti-dsDNA antibodies detected by *Crithidia luciliae* were the most frequently observed autoantibodies (80.5%), followed by anti-SSA antibodies (62.2%). A similar serological profile was observed in patients without a family history of SLE, with anti-dsDNA antibodies detected by *Crithidia luciliae* and anti-SSA antibodies present in 69.6% and 57.5% of patients, respectively. No statistically significant differences were found between the two groups in the prevalence of anti-SSA, anti-SSB, anti-histone, anti-nucleosome, anti-Smith, anti-RNP, or anti-dsDNA antibodies, irrespective of the detection method (all *p* > 0.05; all *q* = 1.000). The titre of anti-dsDNA antibodies measured by *Crithidia luciliae* also did not differ significantly between the groups (*p* = 0.338, *q* = 1.000).

Similarly, the prevalence of antiphospholipid antibodies, including lupus anticoagulant, anti-cardiolipin (IgG/IgM), and anti-β_2_ glycoprotein I (IgG/IgM) antibodies, as well as any antiphospholipid antibody and triple antiphospholipid positivity, was comparable between patients with and without a family history of SLE (all *p* > 0.05; all *q* = 1.000).

### 3.5. Treatment Patterns in Systemic Lupus Erythematosus Patients with Family History of Systemic Lupus Erythematosus

Next, therapeutic approaches were compared between patients with and without a family history of SLE (Table 5). Glucocorticosteroids, administered either orally or intravenously, were the most frequently used treatment in both groups, with comparable rates of use (93.6% in patients with a family history of SLE vs. 96.4% in those without a family history; OR = 0.54, 95% CI: 0.16–2.93; *p* = 0.412; *q* = 1.000). Likewise, the use of chloroquine, hydroxychloroquine, azathioprine, methotrexate, cyclosporine, belimumab, mycophenolate mofetil, cyclophosphamide, rituximab, and intravenous immunoglobulins did not differ significantly between the two groups (all *p* > 0.05; all *q* = 1.000). Anifrolumab was used in 0.8% of patients without a family history of SLE and in none of the patients with a family history, with no significant difference between the groups (*p* = 1.000; *q* = 1.000). Similarly, plasmapheresis was used infrequently in both groups (8.7% vs. 3.8% in patients with and without a family history of SLE, respectively; *p* = 0.111; *q* = 1.000) and did not differ significantly between the groups.

### 3.6. Characteristics of Systemic Lupus Erythematosus Patients with Family History of Systemic Lupus Erythematosus

Among patients with a family history of SLE, affected relatives were classified according to the degree of kinship as first-degree or distant relatives. The Benjamini–Hochberg procedure was applied separately across the 84 comparisons performed in this kinship analysis. The reported familial relationships and the corresponding kinship categories are summarized in Table 6. Several patients reported more than one affected relative; entries listing multiple relatives represent a single patient. The most frequently reported first-degree relatives were sisters (*n* = 9), mothers (*n* = 7), daughters (*n* = 5), brothers (*n* = 4), and sons (*n* = 2). Among more distant relatives, the most frequently reported relationships were aunts (*n* = 3), cousins (*n* = 2), maternal great-aunts (*n* = 2), and nieces (*n* = 2).

Patients with an affected first-degree relative were compared with those with more distant affected relatives. No statistically significant differences were observed in age at first SLE symptom, age at SLE diagnosis, or the distribution of juvenile-, adult-, and late-onset SLE between the two groups (*p* > 0.05). Arthritis was recorded in 26/32 (81.2%) patients with an affected first-degree relative compared with 6/13 (46.2%) patients with more distant affected relatives (*p* = 0.030). In contrast, malar rash was recorded in 10/32 (31.2%) versus 12/14 (85.7%) patients (*p* = 0.001), oral and/or nasal ulcers in 2/32 (6.2%) versus 7/14 (50.0%) patients (*p* = 0.002), and thrombocytopenia in 8/30 (26.7%) versus 10/14 (71.4%) patients (*p* = 0.008), respectively. However, none of these associations remained statistically significant after the separate Benjamini–Hochberg correction for the 84 kinship comparisons (all *q* ≥ 0.068). No other clinically relevant differences were identified between the groups (all *q* > 0.05).

Regarding the autoantibody profile, anti-histone antibodies were detected in 6/32 (18.8%) patients with an affected first-degree relative compared with 7/13 (53.8%) patients with more distant affected relatives (*p* = 0.030; *q* = 0.504). Anti-RNP antibodies were detected in 5/32 (15.6%) versus 6/13 (46.2%) patients, respectively (*p* = 0.053; *q* = 0.562). Neither association remained statistically significant after the separate Benjamini–Hochberg correction for the 84 kinship comparisons. No other significant differences were observed in the analyzed autoantibody profiles (all *p* > 0.05).

Similarly, no statistically significant differences were observed between the first-degree and distant-relative groups with regard to comorbidities, treatment exposure, or mortality (all *q* > 0.05).

## 4. Discussion

The present study evaluated the demographic characteristics, clinical manifestations, comorbidities, laboratory features, and immunosuppressive treatment of patients with SLE according to the presence or absence of a family history of SLE in a large European cohort [15]. Recognition of a family history of SLE may increase clinical awareness among at-risk relatives and facilitate earlier evaluation of symptoms suggestive of SLE. However, our findings do not support a fundamentally different therapeutic approach and should be considered hypothesis-generating.

Among 723 patients with SLE, 47 (6.5%) had a positive family history of SLE. This prevalence is lower than that reported in previous studies, in which the frequency of a family history of SLE ranged from 7.75% to 50.0% across different cohorts [19,20,21]. Such considerable variability is likely attributable to differences in ethnic background, geographic distribution, disease severity, definitions of a family history of SLE, and study design [19,20,21,22,23,24]. Moreover, most published studies have been conducted in Asian or North American populations, whereas data from European cohorts remain limited [19,20,21,22,23,24]. In addition, a family history in our study was obtained primarily from patient interviews and could only be confirmed using medical records in some cases, which may have resulted in underreporting. Nevertheless, our findings confirm that a family history of SLE represents a relatively uncommon subgroup of patients in routine clinical practice.

The female-to-male ratio among patients with a family history of SLE in our cohort was 5.29:1 and did not differ significantly from that observed in patients without family history of SLE. Although this ratio was lower than those reported in previous studies (7:1–15:1), our findings are consistent with the majority of published reports demonstrating no significant sex-related differences between those with and without family history of SLE [15].

Similarly, no significant differences were observed in the age at disease onset, age at first symptoms, or disease duration between patients with and without family history of SLE. These findings are consistent with previous studies reporting comparable ages at disease onset and study inclusion regardless of family history [15]. Likewise, Abutiban et al. found no significant difference in disease duration between those with and without family history of SLE, further supporting our observations [24].

Despite the well-established genetic contribution to SLE susceptibility, the overall clinical profile was largely similar between patients with and without a family history of SLE. Although arthralgia was nominally more frequent among patients with a family history of SLE (97.8% vs. 88.5%, *p* = 0.049), this association did not remain statistically significant after correction for multiple comparisons (*q* = 1.000). Similarly, the difference in overall joint manifestations was not statistically significant (97.9% vs. 88.2%, *p* = 0.051; *q* = 1.000). In contrast to our findings, the meta-analysis by Chen et al. found no significant difference in joint involvement between those with and without family history of the disease. The same meta-analysis demonstrated that SLE patients with a family history of SLE were associated with a lower prevalence of photosensitivity, lupus nephritis, and thrombocytopenia than those without family history of SLE [15]. Taken together, the available evidence does not provide consistent support for a distinct musculoskeletal or overall clinical phenotype associated with a positive family history of SLE.

Stratification according to the degree of kinship revealed several nominal differences between patients with affected first-degree relatives and those with more distant affected relatives. Patients with an affected first-degree relative more frequently presented with arthritis, whereas malar rash, oral and/or nasal ulcers, and thrombocytopenia were more frequently observed among those with more distant affected relatives. However, none of these associations remained statistically significant after the separate Benjamini–Hochberg correction applied to the kinship analysis. Similarly, differences in selected autoantibodies, including anti-histone and anti-RNP antibodies, did not remain statistically significant after the separate Benjamini–Hochberg correction applied to the kinship comparisons. Notably, the lower prevalence of thrombocytopenia among patients with affected first-degree relatives is in line with the findings of the meta-analysis by Chen et al. [15]. Nevertheless, given the small size of the kinship subgroups and the large number of comparisons, these findings should be interpreted cautiously and considered hypothesis-generating rather than evidence of distinct clinical phenotypes according to the degree of familial relatedness. Although conducted in patients with cutaneous lupus erythematosus rather than SLE, Keum et al. also emphasized the predominance of affected first-degree relatives among patients with a positive family history, supporting the importance of considering the degree of kinship in studies of family history of SLE [16].

Consistent with the largely similar clinical phenotype observed in patients with and without family history of SLE, I found no differences in the autoantibody profile, antiphospholipid antibody prevalence, or immunosuppressive treatment patterns between the two groups.

An unexpected finding of the present study was the nominally higher prevalence of myocardial infarction among patients with a family history of SLE (14.9% vs. 5.5%; OR = 3.02, 95% CI: 1.07–7.44; *p* = 0.019). However, this association did not remain statistically significant after correction for multiple comparisons (*q* = 0.912). Cardiovascular disease remains one of the leading causes of morbidity and mortality in SLE and results from a complex interplay of chronic systemic inflammation, endothelial dysfunction, accelerated atherosclerosis, hypercoagulability, antiphospholipid antibodies, traditional cardiovascular risk factors, and the cumulative effects of long-term glucocorticoid therapy [25]. Interestingly, the prevalence of conventional cardiovascular risk factors, including arterial hypertension, diabetes mellitus, hypercholesterolemia, and antiphospholipid antibodies, did not differ between patients with and without family history of SLE in our cohort. Although the observed association may warrant further investigation, the small number of myocardial infarction events, lack of multivariable adjustment, and retrospective design preclude definitive conclusions. Residual confounding by age, disease duration, smoking, cumulative corticosteroid exposure, and other unmeasured or time-dependent factors cannot be excluded. The finding may also represent a chance association related to the limited sample size and multiple comparisons. Further studies in larger, independent cohorts are warranted to determine whether a family history of SLE contributes to cardiovascular risk beyond established risk factors.

Overall, our findings indicate that patients with a positive family history of SLE share most demographic, clinical, serological, and therapeutic characteristics with patients without a family history of SLE and do not appear to constitute a distinct clinical phenotype after correction for multiple comparisons. The nominal associations observed for selected clinical manifestations and myocardial infarction, as well as the differences observed according to the degree of kinship, should not be interpreted as definitive evidence of familial clinical phenotypes. Rather, these findings may generate hypotheses regarding whether specific manifestations or comorbidities cluster within familial SLE. In particular, the degree of kinship should be considered in future studies, but its interpretation should account for the fact that kinship is also a proxy for shared environmental factors, healthcare-seeking behaviour, diagnostic awareness, and ascertainment, rather than a direct measure of genetic susceptibility.

Further multicenter studies involving larger, ethnically diverse cohorts and integrating comprehensive clinical phenotyping with genetic and genomic analyses are needed to determine whether the nominal associations observed in the present study are reproducible and to clarify the contribution of inherited factors to disease susceptibility and clinical heterogeneity among patients with a family history of SLE.

This study has several limitations. First, its retrospective single-center design is inherently susceptible to selection and information bias and may limit the generalizability of the findings to other populations. Second, the relatively small number of patients with a positive family history of SLE, particularly within the subgroup analyses according to the degree of kinship, reduced the statistical power to detect less frequent clinical associations. Third, family history was based on information obtained from medical records and/or patient self-report, introducing the possibility of underreporting and misclassification. No genetic testing was performed to confirm a hereditary or monogenic basis of SLE; therefore, the observed associations should be interpreted as associations between family history and clinical phenotype rather than genotype–phenotype associations. Furthermore, the degree of kinship represents only a crude proxy for shared genetic susceptibility and may also reflect shared environmental factors, healthcare-seeking behaviour, diagnostic awareness, and ascertainment. Finally, the lack of patient-reported outcome measures, such as health-related quality of life or disease burden, limited the assessment of the broader clinical impact of a positive family history of SLE. Disease activity, flare burden, accumulated organ damage, hospitalizations, and treatment response were also not systematically assessed. The large number of comparisons may have increased the likelihood of chance findings, and the results of the subgroup analyses should therefore be interpreted cautiously, particularly given the limited sample size. Given the observational nature of the study, the identified associations should be considered hypothesis-generating and require confirmation in larger prospective multicenter cohorts.

## 5. Conclusions

A positive family history of SLE was reported in a small proportion of patients in this European cohort and was associated with demographic, clinical, serological, and treatment profiles largely comparable to those of patients without a family history of SLE. Although arthralgia and overall joint involvement were nominally more frequent among patients with a positive family history of SLE, these associations did not remain significant after correction for multiple comparisons. Similarly, several differences observed according to the degree of kinship did not remain statistically significant after correction. The nominal association between a positive family history of SLE and myocardial infarction also requires confirmation in larger, adequately powered studies. Overall, our findings do not support a distinct clinical phenotype associated with a positive family history of SLE. The observed nominal associations should be considered hypothesis-generating and warrant confirmation in larger prospective multicenter cohorts, ideally incorporating genetic data and systematic assessment of relevant clinical and cardiovascular factors.

## Figures and Tables

**Table 1:** Demographic characteristics and vital status by familial systemic lupus erythematosus status.

Characteristic	Sporadic (*n* = 676)	Familial (*n* = 47)	Odds Ratio (95% CI)	Difference, pp (95% CI)	*p*	*q*
Female sex	595/676 (88.0%)	40/47 (85.1%)	0.78 (0.33–2.13)	−2.9 (−13.4 to 7.6)	0.495	1.000
Current age, years	48 (38–60); *n* = 578	48 (36–59); *n* = 37	—	—	0.751	1.000
Age at first SLE symptom, years	31 (23–45); *n* = 667	32 (20–44.5); *n* = 47	—	—	0.598	1.000
Age at SLE diagnosis, years	33.5 (25–47); *n* = 666	33 (21.5–50); *n* = 47	—	—	0.826	1.000
Diagnostic delay, years	0 (0–2); *n* = 676	0 (0–2); *n* = 47	—	—	0.979	1.000
Death	42/618 (6.8%)	3/40 (7.5%)	1.11 (0.21–3.75)	0.7 (−7.7 to 9.1)	0.749	1.000

Note. Values are *n*/N (%) or median (Q1–Q3). *p*: Fisher exact or Wilcoxon rank-sum test; *q*: Benjamini–Hochberg adjusted across 97 primary comparisons. Differences are familial minus sporadic percentage points. For mortality, vital status was available for 618 sporadic and 40 familial patients. The smaller denominators reflect varying patterns of data availability in the registry.

**Table 2:** Clinical manifestations by familial systemic lupus erythematosus status.

**Characteristic**	**Sporadic** **(*n* = 676)**	**Familial** **(*n* = 47)**	**Odds Ratio** **(95% CI)**	**Difference, pp** **(95% CI)**	***p***	***q***
Constitutional symptoms	553/676 (81.8%)	41/47 (87.2%)	1.52 (0.62–4.48)	5.4 (−4.5 to 15.4)	0.433	1.000
Fever	294/660 (44.5%)	23/45 (51.1%)	1.30 (0.68–2.50)	6.6 (−8.5 to 21.7)	0.440	1.000
Fatigue or weakness	454/660 (68.8%)	32/46 (69.6%)	1.04 (0.52–2.15)	0.8 (−13.0 to 14.5)	1.000	1.000
Myalgia	258/661 (39.0%)	17/45 (37.8%)	0.95 (0.48–1.84)	−1.3 (−15.9 to 13.4)	1.000	1.000
Weight loss	163/661 (24.7%)	13/45 (28.9%)	1.24 (0.58–2.50)	4.2 (−9.4 to 17.9)	0.593	1.000
Lymphadenopathy	141/662 (21.3%)	13/45 (28.9%)	1.50 (0.70–3.03)	7.6 (−6.0 to 21.2)	0.262	1.000
Mucocutaneous manifestations	549/676 (81.2%)	33/47 (70.2%)	0.55 (0.27–1.14)	−11.0 (−24.4 to 2.4)	0.084	1.000
Malar rash	312/672 (46.4%)	22/46 (47.8%)	1.06 (0.55–2.01)	1.4 (−13.5 to 16.3)	0.880	1.000
Discoid rash	65/672 (9.7%)	5/46 (10.9%)	1.14 (0.34–3.02)	1.2 (−8.1 to 10.5)	0.796	1.000
Urticaria	55/672 (8.2%)	2/46 (4.3%)	0.51 (0.06–2.05)	−3.8 (−10.1 to 2.4)	0.570	1.000
Cutaneous vasculitis	37/672 (5.5%)	4/46 (8.7%)	1.63 (0.40–4.87)	3.2 (−5.1 to 11.5)	0.325	1.000
Alopecia	172/672 (25.6%)	15/46 (32.6%)	1.41 (0.69–2.76)	7.0 (−6.9 to 21.0)	0.300	1.000
Oral and/or nasal ulcers	109/672 (16.2%)	9/46 (19.6%)	1.26 (0.52–2.75)	3.3 (−8.5 to 15.1)	0.539	1.000
Other skin changes	453/673 (67.3%)	30/46 (65.2%)	0.91 (0.47–1.83)	−2.1 (−16.3 to 12.1)	0.748	1.000
Joint manifestations (recorded any_joint)	596/676 (88.2%)	46/47 (97.9%)	6.17 (1.02–252)	9.7 (4.9 to 14.5)	0.051	1.000
Arthritis	399/669 (59.6%)	32/45 (71.1%)	1.66 (0.83–3.52)	11.5 (−2.3 to 25.2)	0.156	1.000
Arthralgia	595/672 (88.5%)	45/46 (97.8%)	5.82 (0.96–238)	9.3 (4.4 to 14.1)	0.049	1.000
Pleural effusion	131/673 (19.5%)	9/47 (19.1%)	0.98 (0.41–2.13)	−0.3 (−12.0 to 11.3)	1.000	1.000
Pericardial effusion	114/664 (17.2%)	6/46 (13.0%)	0.72 (0.25–1.77)	−4.1 (−14.3 to 6.0)	0.548	1.000
Pericarditis	26/674 (3.9%)	2/47 (4.3%)	1.11 (0.12–4.67)	0.4 (−5.6 to 6.3)	0.703	1.000
Haematological manifestations	628/676 (92.9%)	41/47 (87.2%)	0.52 (0.21–1.58)	−5.7 (−15.4 to 4.1)	0.152	1.000
Leucopenia	412/665 (62.0%)	26/45 (57.8%)	0.84 (0.44–1.64)	−4.2 (−19.1 to 10.7)	0.635	1.000
Lymphopenia	*n* = 652	*n* = 44	—	—	0.629	1.000
None	113/652 (17.3%)	10/44 (22.7%)				
1000–1500/µL	384/652 (58.9%)	25/44 (56.8%)				
<1000/µL	155/652 (23.8%)	9/44 (20.5%)				
Anaemia	509/666 (76.4%)	32/46 (69.6%)	0.71 (0.36–1.47)	−6.9 (−20.5 to 6.8)	0.288	1.000
Haemolytic anaemia	76/271 (28.0%)	4/15 (26.7%)	0.93 (0.21–3.27)	−1.4 (−24.4 to 21.6)	1.000	1.000
Thrombocytopenia	206/664 (31.0%)	18/44 (40.9%)	1.54 (0.78–2.99)	9.9 (−5.1 to 24.8)	0.183	1.000
Macrophage activation syndrome	8/674 (1.2%)	0/46 (0.0%)	0.00 (0.00–8.76)	−1.2 (−2.0 to −0.4)	1.000	1.000
Thrombotic thrombocytopenic purpura	2/676 (0.3%)	0/47 (0.0%)	0.00 (0.00–77.1)	−0.3 (−0.7 to 0.1)	1.000	1.000
Kidney involvement	252/673 (37.4%)	20/47 (42.6%)	1.24 (0.64–2.34)	5.1 (−9.5 to 19.7)	0.534	1.000
Lupus nephritis class (ISN/RPS)	*n* = 139	*n* = 13	—	—	0.010	0.912
I	1/139 (0.7%)	2/13 (15.4%)				
II	25/139 (18.0%)	4/13 (30.8%)				
III	17/139 (12.2%)	2/13 (15.4%)				
IV	74/139 (53.2%)	4/13 (30.8%)				
V	19/139 (13.7%)	0/13 (0.0%)				
VI	3/139 (2.2%)	1/13 (7.7%)				
Proteinuria > 0.5 g/day	239/658 (36.3%)	16/45 (35.6%)	0.97 (0.48–1.88)	−0.8 (−15.2 to 13.7)	1.000	1.000
Proteinuria > 3.5 g/day	134/631 (21.2%)	8/42 (19.0%)	0.87 (0.34–1.98)	−2.2 (−14.5 to 10.1)	0.847	1.000
Urinary casts	121/591 (20.5%)	9/42 (21.4%)	1.06 (0.43–2.34)	1.0 (−11.9 to 13.8)	0.845	1.000
Erythrocyturia	197/630 (31.3%)	14/42 (33.3%)	1.10 (0.52–2.21)	2.1 (−12.6 to 16.8)	0.864	1.000
Leucocyturia	232/645 (36.0%)	14/43 (32.6%)	0.86 (0.41–1.72)	−3.4 (−17.9 to 11.1)	0.744	1.000
Neurological signs	85/676 (12.6%)	6/47 (12.8%)	1.02 (0.34–2.51)	0.2 (−9.7 to 10.1)	1.000	1.000
Central nervous system involvement	58/674 (8.6%)	6/46 (13.0%)	1.59 (0.53–4.00)	4.4 (−5.5 to 14.4)	0.286	1.000
Peripheral nervous system involvement	39/674 (5.8%)	1/46 (2.2%)	0.36 (0.01–2.25)	−3.6 (−8.2 to 1.0)	0.505	1.000
Lung involvement	66/676 (9.8%)	6/47 (12.8%)	1.35 (0.45–3.37)	3.0 (−6.8 to 12.8)	0.454	1.000
Interstitial lung disease	43/675 (6.4%)	3/47 (6.4%)	1.00 (0.19–3.33)	0.0 (−7.2 to 7.2)	1.000	1.000
Diffuse alveolar haemorrhage	10/675 (1.5%)	1/47 (2.1%)	1.44 (0.03–10.6)	0.6 (−3.6 to 4.9)	0.526	1.000
Pulmonary hypertension	25/662 (3.8%)	2/47 (4.3%)	1.13 (0.13–4.79)	0.5 (−5.5 to 6.4)	0.698	1.000
Lupoid hepatitis	37/675 (5.5%)	1/46 (2.2%)	0.38 (0.01–2.39)	−3.3 (−7.9 to 1.2)	0.503	1.000

Note. Values are *n*/N (%) or median (Q1–Q3). *p*: Fisher exact or Wilcoxon rank-sum test; *q*: Benjamini–Hochberg adjusted across 97 primary comparisons. Differences are familial minus sporadic percentage points. For haemolytic anaemia, data were available for 271 sporadic and 15 familial patients. The smaller denominators reflect varying patterns of data availability in the registry.

**Table 3:** Comorbidities by familial systemic lupus erythematosus status.

Characteristic	Sporadic (*n* = 676)	Familial (*n* = 47)	Odds Ratio (95% CI)	Difference, pp (95% CI)	*p*	*q*
Hypothyroidism	172/676 (25.4%)	11/47 (23.4%)	0.90 (0.40–1.85)	−2.0 (−14.6 to 10.5)	0.863	1.000
Hyperthyroidism	36/676 (5.3%)	4/47 (8.5%)	1.65 (0.41–4.93)	3.2 (−5.0 to 11.3)	0.321	1.000
Arterial hypertension	384/676 (56.8%)	29/47 (61.7%)	1.22 (0.64–2.39)	4.9 (−9.5 to 19.3)	0.545	1.000
Diabetes mellitus	81/676 (12.0%)	6/47 (12.8%)	1.07 (0.36–2.66)	0.8 (−9.1 to 10.6)	0.818	1.000
Hypercholesterolaemia	355/676 (52.5%)	26/47 (55.3%)	1.12 (0.59–2.14)	2.8 (−11.9 to 17.5)	0.764	1.000
Ever smoker	217/633 (34.3%)	17/40 (42.5%)	1.42 (0.69–2.84)	8.2 (−7.5 to 24.0)	0.307	1.000
Heart failure	42/675 (6.2%)	2/47 (4.3%)	0.67 (0.08–2.73)	−2.0 (−8.0 to 4.1)	1.000	1.000
Atrial fibrillation	30/676 (4.4%)	1/47 (2.1%)	0.47 (0.01–2.96)	−2.3 (−6.7 to 2.1)	0.714	1.000
Peripheral artery disease	38/676 (5.6%)	3/47 (6.4%)	1.14 (0.22–3.84)	0.8 (−6.4 to 8.0)	0.744	1.000
End-stage kidney disease	23/674 (3.4%)	1/47 (2.1%)	0.62 (0.01–3.97)	−1.3 (−5.6 to 3.1)	1.000	1.000
MGUS	18/676 (2.7%)	2/47 (4.3%)	1.62 (0.18–7.13)	1.6 (−4.3 to 7.5)	0.378	1.000
Malignancy	73/675 (10.8%)	6/47 (12.8%)	1.21 (0.40–2.99)	2.0 (−7.9 to 11.8)	0.630	1.000
Stroke	56/676 (8.3%)	4/47 (8.5%)	1.03 (0.26–2.99)	0.2 (−8.0 to 8.5)	1.000	1.000
Transient ischaemic attack	11/676 (1.6%)	2/47 (4.3%)	2.68 (0.28–12.9)	2.6 (−3.2 to 8.5)	0.205	1.000
Myocardial infarction (lifetime)	37/676 (5.5%)	7/47 (14.9%)	3.02 (1.07–7.44)	9.4 (−0.9 to 19.7)	0.019	0.912
Other arterial thrombotic episodes	19/676 (2.8%)	1/47 (2.1%)	0.75 (0.02–4.95)	−0.7 (−5.0 to 3.6)	1.000	1.000
Deep venous thrombosis	107/675 (15.9%)	5/47 (10.6%)	0.63 (0.19–1.65)	−5.2 (−14.4 to 4.0)	0.410	1.000
Pulmonary embolism	29/675 (4.3%)	2/47 (4.3%)	0.99 (0.11–4.13)	−0.0 (−6.0 to 5.9)	1.000	1.000
Deep venous thrombosis and pulmonary embolism	15/676 (2.2%)	2/47 (4.3%)	1.96 (0.21–8.83)	2.0 (−3.8 to 7.9)	0.304	1.000
Other venous thrombotic episodes	16/676 (2.4%)	0/47 (0.0%)	0.00 (0.00–3.78)	−2.4 (−3.5 to −1.2)	0.616	1.000

Note. Values are *n*/N (%) or median (Q1–Q3). *p*: Fisher exact or Wilcoxon rank-sum; *q*: Benjamini–Hochberg adjusted across 97 comparisons. Differences are familial minus sporadic percentage points.

**Table 4:** Autoantibody profiles by familial systemic lupus erythematosus status.

Characteristic	Sporadic (*n* = 676)	Familial (*n* = 47)	Odds Ratio (95% CI)	Difference, pp (95% CI)	*p*	*q*
ANA titre (recorded reciprocal titre)	10,240 (2560–20,480); *n* = 665	10,240 (2560–20,480); *n* = 46	—	—	0.962	1.000
Anti-SSA antibodies	361/628 (57.5%)	28/45 (62.2%)	1.22 (0.63–2.42)	4.7 (−9.9 to 19.4)	0.640	1.000
Anti-SSB antibodies	174/628 (27.7%)	16/45 (35.6%)	1.44 (0.71–2.82)	7.8 (−6.6 to 22.3)	0.303	1.000
Anti-histone antibodies	179/628 (28.5%)	13/45 (28.9%)	1.02 (0.48–2.05)	0.4 (−13.3 to 14.1)	1.000	1.000
Anti-nucleosome antibodies	226/627 (36.0%)	17/45 (37.8%)	1.08 (0.54–2.09)	1.7 (−12.9 to 16.4)	0.873	1.000
Anti-Smith antibodies	81/625 (13.0%)	5/45 (11.1%)	0.84 (0.25–2.22)	−1.8 (−11.4 to 7.7)	1.000	1.000
Anti-RNP antibodies	145/626 (23.2%)	11/45 (24.4%)	1.07 (0.48–2.24)	1.3 (−11.7 to 14.3)	0.855	1.000
Anti-dsDNA antibodies (immunoblot)	246/624 (39.4%)	20/45 (44.4%)	1.23 (0.63–2.36)	5.0 (−10.0 to 20.0)	0.531	1.000
Anti-dsDNA antibodies (*Crithidia luciliae*)	413/593 (69.6%)	33/41 (80.5%)	1.80 (0.79–4.59)	10.8 (−1.8 to 23.5)	0.160	1.000
Anti-dsDNA titre (*Crithidia luciliae*)	80 (0–320); *n* = 588	80 (12.5–320); *n* = 42	—	—	0.338	1.000
Lupus anticoagulant	135/493 (27.4%)	9/40 (22.5%)	0.77 (0.31–1.71)	−4.9 (−18.4 to 8.6)	0.582	1.000
Anti-cardiolipin antibodies IgG and/or IgM	302/532 (56.8%)	18/42 (42.9%)	0.57 (0.28–1.13)	−13.9 (−29.5 to 1.6)	0.106	1.000
Anti-beta2 glycoprotein I IgG and/or IgM	108/449 (24.1%)	9/34 (26.5%)	1.14 (0.45–2.61)	2.4 (−12.9 to 17.8)	0.835	1.000
Any antiphospholipid antibody	332/487 (68.2%)	20/38 (52.6%)	0.52 (0.25–1.07)	−15.5 (−31.9 to 0.9)	0.071	1.000
Triple antiphospholipid positivity	67/676 (9.9%)	6/47 (12.8%)	1.33 (0.44–3.31)	2.9 (−6.9 to 12.7)	0.460	1.000

Note. Values are *n*/N (%) or median (Q1–Q3). *p*: Fisher exact or Wilcoxon rank-sum; *q*: Benjamini–Hochberg adjusted across 97 comparisons. Differences are familial minus sporadic percentage points.

**Table 5:** Treatment exposure by familial systemic lupus erythematosus status.

Characteristic	Sporadic (*n* = 676)	Familial (*n* = 47)	Odds Ratio (95% CI)	Difference, pp (95% CI)	*p*	*q*
Glucocorticosteroids (oral/intravenous)	648/672 (96.4%)	44/47 (93.6%)	0.54 (0.16–2.93)	−2.8 (−9.9 to 4.3)	0.412	1.000
Chloroquine	339/668 (50.7%)	27/46 (58.7%)	1.38 (0.72–2.68)	7.9 (−6.8 to 22.7)	0.361	1.000
Hydroxychloroquine	369/668 (55.2%)	27/46 (58.7%)	1.15 (0.60–2.24)	3.5 (−11.3 to 18.2)	0.759	1.000
Azathioprine	272/668 (40.7%)	19/46 (41.3%)	1.02 (0.53–1.96)	0.6 (−14.1 to 15.3)	1.000	1.000
Methotrexate	150/666 (22.5%)	8/46 (17.4%)	0.72 (0.29–1.62)	−5.1 (−16.5 to 6.3)	0.470	1.000
Cyclosporine	65/665 (9.8%)	5/46 (10.9%)	1.13 (0.34–2.99)	1.1 (−8.2 to 10.4)	0.797	1.000
Belimumab	29/663 (4.4%)	4/46 (8.7%)	2.08 (0.51–6.33)	4.3 (−4.0 to 12.6)	0.263	1.000
Mycophenolate mofetil	236/664 (35.5%)	19/46 (41.3%)	1.28 (0.66–2.44)	5.8 (−8.9 to 20.4)	0.431	1.000
Cyclophosphamide	214/665 (32.2%)	15/46 (32.6%)	1.02 (0.50–2.00)	0.4 (−13.6 to 14.4)	1.000	1.000
Rituximab	30/663 (4.5%)	0/46 (0.0%)	0.00 (0.00–1.88)	−4.5 (−6.1 to −2.9)	0.251	1.000
Immunoglobulins	27/664 (4.1%)	1/46 (2.2%)	0.52 (0.01–3.34)	−1.9 (−6.4 to 2.6)	1.000	1.000
Plasmapheresis	25/663 (3.8%)	4/46 (8.7%)	2.43 (0.59–7.50)	4.9 (−3.3 to 13.2)	0.111	1.000
Anifrolumab	5/664 (0.8%)	0/46 (0.0%)	0.00 (0.00–16.0)	−0.8 (−1.4 to −0.1)	1.000	1.000

Note. Values are *n*/N (%) or median (Q1–Q3). *p*: Fisher exact or Wilcoxon rank-sum; *q*: Benjamini–Hochberg adjusted across 97 comparisons. Differences are familial minus sporadic percentage points.

**Table 6:** Reported familial relationships by kinship category.

Kinship Group	Relationship as Recorded in Workbook	Patients, *n*
**First-degree relatives (*n* = 33)**		
	sister	9
	mother	7
	daughter	5
	brother	4
	son	2
	child, child	1
	mother, sister	1
	sister, grandmother	1
	sister, daughter, daughter	1
	father	1
	father, uncle (father’s brother), uncle (father’s brother), aunt (father’s sister), female cousin	1
**Distant relatives (*n* = 14)**		
	aunt	3
	female cousin	2
	aunt	2
	niece (sister’s daughter)	2
	niece (brother’s daughter)	1
	cousin’s daughter	1
	two female cousins	1
	father’s cousin, uncle (father’s brother)	1
	mother’s cousin	1

Note. Entries listing multiple relatives represent one patient.

## Data Availability

The data presented in this study are available on reasonable request from: the corresponding author.

## Abbreviations

The following abbreviations are used in this manuscript:

ACR	American College of Rheumatology
ANA	Anti-nuclear antibodies
dsDNA	Double-stranded DNA
ELISA	Enzyme-linked immunosorbent assay
ESKD	End-stage kidney disease
EULAR	The European Alliance of Associations for Rheumatology
GWAS	Genome-wide association studies
IIF	Indirect immunofluorescence
MGUS	Monoclonal gammopathy of undetermined significance
MHC	Major histocompatibility complex
RNP	Ribonucleoprotein
SSA	Sjögren’s syndrome-related antigen A
SSB	Sjögren’s syndrome-related antigen B
SLE	Systemic lupus erythematosus
